# Radiographically Occult and Subtle Fractures: A Pictorial Review

**DOI:** 10.1155/2013/370169

**Published:** 2013-03-17

**Authors:** Mohamed Jarraya, Daichi Hayashi, Frank W. Roemer, Michel D. Crema, Luis Diaz, Jane Conlin, Monica D. Marra, Nabil Jomaah, Ali Guermazi

**Affiliations:** ^1^Department of Radiology, Musculoskeletal Section, Boston University School of Medicine, 820 Harrison Avenue, FGH Building, 3rd Floor, Boston, MA 02118, USA; ^2^Department of Radiology, University of Erlangen, 91054 Erlangen, Germany; ^3^Department of Radiology, Hospital do Coração sp (HCor) and Teleimagem, 04004-030 São Paulo, SP, Brazil; ^4^Department of Radiology, VA Boston Healthcare System, Boston, MA 02130, USA; ^5^Department of Radiology, Seattle Cancer Care Alliance, University of Washington, Seattle, WA 98109, USA; ^6^Department of Radiology, Aspetar, Qatar Orthopaedic and Sports Medicine Hospital, P.O. Box 29222, Doha, Qatar

## Abstract

Radiographically occult and subtle fractures are a diagnostic challenge. They may be divided into (1) “high energy trauma fracture,” (2) “fatigue fracture” from cyclical and sustained mechanical stress, and (3) “insufficiency fracture” occurring in weakened bone (e.g., in osteoporosis and postradiotherapy). Independently of the cause, the initial radiographic examination can be negative either because the findings seem normal or are too subtle. Early detection of these fractures is crucial to explain the patient's symptoms and prevent further complications. Advanced imaging tools such as computed tomography, magnetic resonance imaging, and scintigraphy are highly valuable in this context. Our aim is to raise the awareness of radiologists and clinicians in these cases by presenting illustrative cases and a discussion of the relevant literature.

## 1. Introduction

Radiographically occult and subtle fractures are a common diagnostic challenge in daily practice. Indeed, fractures represent up to 80% of the missed diagnoses in the emergency department [1]. Failure to recognize the subtle signs of osseous injury is one of the reasons behind this major diagnostic challenge [2]. While occult fractures present no radiographic findings, radiographically subtle fractures are easily overlooked on initial radiographs. In both cases, a negative radiographic diagnosis with prominent clinical suspicion of osseous injury will prompt advanced imaging examination such as computed tomography (CT), magnetic resonance imaging (MRI), ultrasound, and nuclear medicine to confirm or exclude the clinically suspected diagnosis. The burden entailed in missing these fractures includes prolonged pain with a loss of function, and disability. Early detection, on the other hand, enables more effective treatment, a shorter hospitalization period if necessary, and decreased medical costs in the long run. It will also prevent inherent complications such as nonunion, malunion, premature osteoarthritis, and avascular osteonecrosis (as in scaphoid fracture) [3]. Occult and subtle fractures may be divided into: (1) fractures associated with high energy trauma; (2) fatigue fracture secondary to repetitive and unusual stress being applied to bone with normal elastic resistance; and (3) insufficiency fracture resulting from normal or minimal stress on a bone with decreased elastic resistance [4]. The term “stress fracture” is more general and encompasses both of the latter two entities [5]. Pediatric and microtrabecular fractures—known as bone bruises and contusions—are outside the scope of this paper. Our aim is to raise the awareness of both clinicians and radiologists to this common problem by illustrating various cases of radiographically occult and subtle fractures.

## 2. Imaging Tools

Thanks to rapid technological advancement, new and more efficient imaging hardware is constantly released for all imaging modalities including CT, MRI, nuclear medicine, and ultrasound. Nonetheless, not every department can afford all new technologies, and radiologists sometimes have to face the challenge of providing the highest diagnostic performance with basic imaging tools. This can only be achieved by ensuring high quality of examination with the available imaging tools.

### 2.1. Conventional Radiographs

Radiography is the first step for detection of fractures. The detection of subtle signs of fracture requires a high standard for the acquisition technique and a thorough and systematic interpretation of radiographic images. Correct diagnosis primarily relies on the reader's experience. Awareness of normal anatomic features is crucial for the interpreter to be able to detect subtle signs of fracture. Fat pads should be carefully examined for convexity, which implies joint effusion (e.g., in the hip and elbow joints). However, the radiographic technique (positioning in particular) must be optimal for this evaluation to be valid [6]. Osseous lines should be checked for integrity (e.g., acetabular rim in the hip). Trabecular angulation, impaction lines, and sclerotic bands also suggest fracture in osseous structures with a significant proportion of cancellous bone such as proximal femur [6].

The general rule is to perform two orthogonal views, but more specific views should be added if there is any suspicion of fracture. Moreover, one should be aware of the commonly encountered lesions and their locations. In wrist trauma, for instance, the interpreter should pay close attention to the scaphoid and triquetrum, which are the two most commonly injured carpal bones [3]. The mechanism of trauma may also be helpful to locate the potential fracture. A fall on an outstretched hand suggests scaphoid fracture. Although the classical presentation consists of a radiolucent line and cortical disruption, the radiographic signs will depend upon the time elapsed between the first clinical symptoms and the time of radiographic examination, the location of the fracture within the bone, and the ratio of cortical to cancellous bone. Particular attention should be paid when analysing the subchondral plate, which may be disrupted or deformed. In metaphyseal areas, delayed signs of fracture include a band of sclerosis perpendicular to the trabeculae, while diaphyseal fractures may present as periosteal thickening [7].

Digital radiography known as tomosynthesis has been shown to be superior to conventional radiographs in the detection of occult fracture of the scaphoid [8]. Tomosynthesis has the ability to demonstrate cortical, as well as moderately displaced trabecular fractures. Thus, the performance of tomosynthesis in detecting radiographically occult fractures is considered as comparable to CT.

### 2.2. Computed Tomography

Multidetector computed tomography (MDCT) is a highly valuable imaging tool for the diagnosis of occult fractures. CT has several advantages including short acquisition time (compared to MRI), the ability to acquire volumetric and isotropic image data sets, the opportunity to reconstruct multiplanar reformations in any arbitrary plane, and excellent spatial resolution [9]. Moreover, the image quality for multiplanar reconstruction may be increased by reducing slice thickness and acquisition pitch. In general, bony structures are best demonstrated by using a small focal spot and using a “bone” algorithm. CT contributes much to the diagnosis of occult fractures by depicting subtle fracture lines, depressed, or distracted articular surfaces and by assessing bone loss [9]. It also detects late bony changes such as increased medullary density, endosteal sclerosis, sclerotic lines in trabecular bone, and periosteal thickening. Moreover, CT aids in excluding other differential diagnoses, especially in case of isolated bone marrow edema, by confirming the normal appearance of the remaining trabeculae and excluding space-occupying lesions such as malignancy and osteomyelitis [10]. 

Newest generation of CT, such as dedicated cone-beam CT (CBCT) system for musculoskeletal extremities, may be beneficial in various conditions, such as arthritis and occult fractures [11]. Although dedicated CBCT for musculoskeletal extremities is still a matter of research, it has been shown to be of potential benefit as an adjunct for CT and MRI. It offers the possibility of volumetric imaging, which may be helpful in case of suspected occult fractures [8]. It also provides higher spatial resolution and a potentially reduced dose compared to CT [11].

### 2.3. Magnetic Resonance Imaging

The diagnostic performance of MRI in the detection of occult fractures has been shown to be comparable [12], or better [13–17] than MDCT. Indeed, while the specificity of both CT and MRI for the diagnosis of fracture can be as high as 100% [18], the sensitivity was reported to be higher for MRI [13–16]. The superiority of MRI over any other imaging modality including MDCT for the detection of occult hip fractures is now recognised [13–15]. For instance, an occult intertrochanteric extension of a greater trochanter fracture can be most effectively appreciated on MRI [19]. Moreover, MRI is extremely helpful in detecting associated soft tissue abnormalities, especially ligamentous lesions [20]. MRI is now considered as the standard in this context [21]. However, because of its relative unavailability in emergency settings and high costs, MRI may only be performed in “high risk patients” with negative X-rays. For example, when a hip occult fracture is suspected, patients with reduced baseline mobility and pain on axial compression are considered at risk and, therefore, should be examined by MRI [22]. MRI signs of occult fractures are evident several weeks before radiographic signs appear [15, 16]. In the hip, a limited and cost effective MR protocol, with only T1 weighted (*W*) coronal images, may enable a reliable diagnosis or exclusion of occult fracture in very little time, for example, 7 minutes [23]. Typically, a linear hypointensity is observed on T1 W images. MRI is also highly sensitive to marrow abnormalities surrounding the fracture line, which appear as hypointensity on T1 W images and hyperintensity on fluid-sensitive sequences [24]. Such signal changes are thought to be a combination of bone marrow edema, intraosseous haemorrhage, and/or granulation tissue [20] and help to identify even undisplaced fractures [25]. However, in the absence of a history of trauma and linear hypointensities on T1 W images, isolated bone marrow edema may represent other pathologies such as osteoid osteoma and sclerosing osteomyelitis [26].

Although 1.5 T and 3 T MR is considered as the current gold standard for the detection of radiographically occult fractures, ultra-high field MR provide higher signal-to-noise ratio and, therefore, is expected to be superior to 1.5 T and 3 T [27]. Ultra-high field MR seems to be promising in the diagnosis of a variety of musculoskeletal conditions including trauma, but it is not used in daily routine yet. 

### 2.4. Nuclear Medicine

The most traditional method is bone scintigraphy. Although scintigraphy is highly sensitive for the detection of occult fracture, its lack of specificity limits its diagnostic utility. However, when MRI is not available, scintigraphy may be of value, especially in the absence of trauma history, for example, for detection of insufficiency and fatigue fractures. While radiography may show only late signs of bone reaction (such as periosteal thickening and band of sclerosis), scintigraphic examination allows for earlier detection of bony changes. Regarding Fluorine-18 2-deoxy-D-glucose (FDG) positron emission tomography (PET), it is critical to be aware that occult fractures may be responsible of marked metabolic uptake, and, thus, represent a potential false positive of metastatic disease [28]. Integrated hybrid single-photon emission computerized tomography (SPECT)/CT combines the detection of abnormal bone metabolism with SPECT, to the precise anatomical detail provided by high resolution CT. For instance, SPECT/CT may be interesting in the detection of radiographic occult fractures of the wrist [29] and other sport-related injuries [30].

### 2.5. Ultrasonography

High frequency ultrasound has been shown to be of value, particularly in the pediatric population [31, 32]. In this case, and in an emergency setting, ultrasonography can be more accessible and less time consuming than radiographs and has high specificity and sensitivity in the evaluation of suspected long bone fractures [32]. The utility of ultrasonography has also been shown for adults with suspicion of wrist trauma [33] or fatigue/stress fracture [34]. Recently, it has been suggested that therapeutic ultrasound may be beneficial as a primary evaluation of bone stress injury [35]; however, its benefit seems to be more evident in selected high risk patients rather than general population [36]. 

## 3. High Energy Trauma Fractures

**Figure 1:**
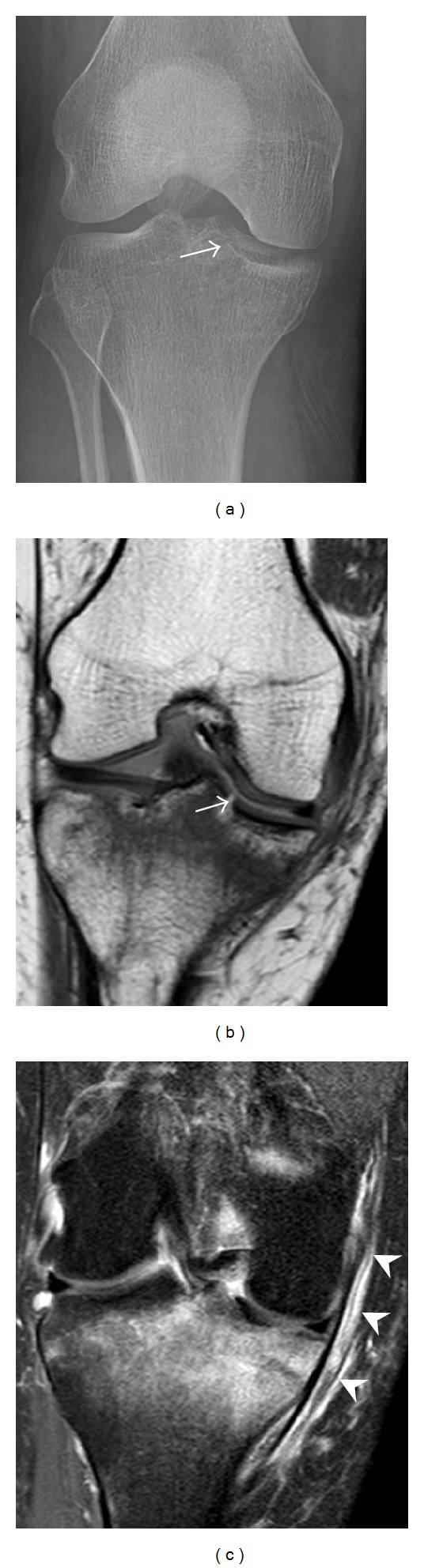
A 56-year-old woman presenting with left knee pain after a fall. (a) Initial anteroposterior radiograph was considered normal, however, subtle cortical disruption of the anterior rim of the medial tibial plateau, medial to the tibial spine, is noted (arrow). (b) Coronal T1-weighted MRI confirms the cortical disruption (arrow) and shows extensive fracture through the proximal tibia. (c) Coronal proton density-weighted image with fat saturation shows extensive edema in the subchondral bone. Note also hypersignal adjacent to the medial collateral ligament corresponding to a grade I sprain (arrowheads).

**Figure 2:**
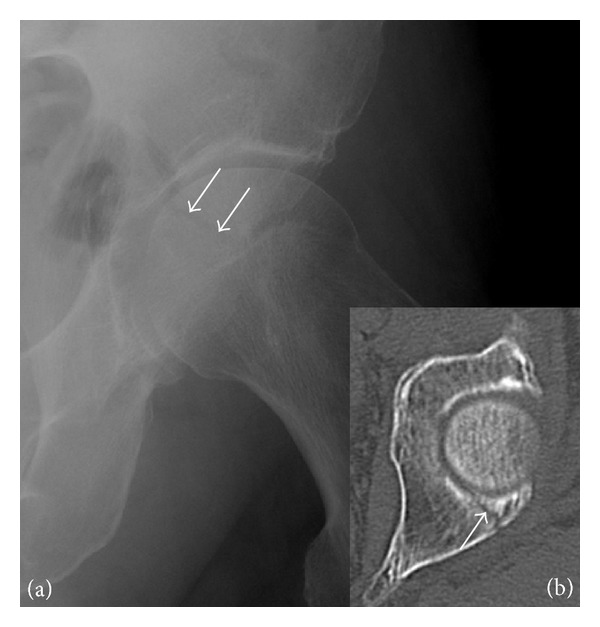
Posterior acetabular fracture in a 49-year-old woman presenting with hip pain after a fall. (a) Anteroposterior radiograph of the left hip shows a radiolucent line through the posterior acetabular wall (arrows). (b) Axial CT confirms the acetabular fracture (arrow).

**Figure 3:**
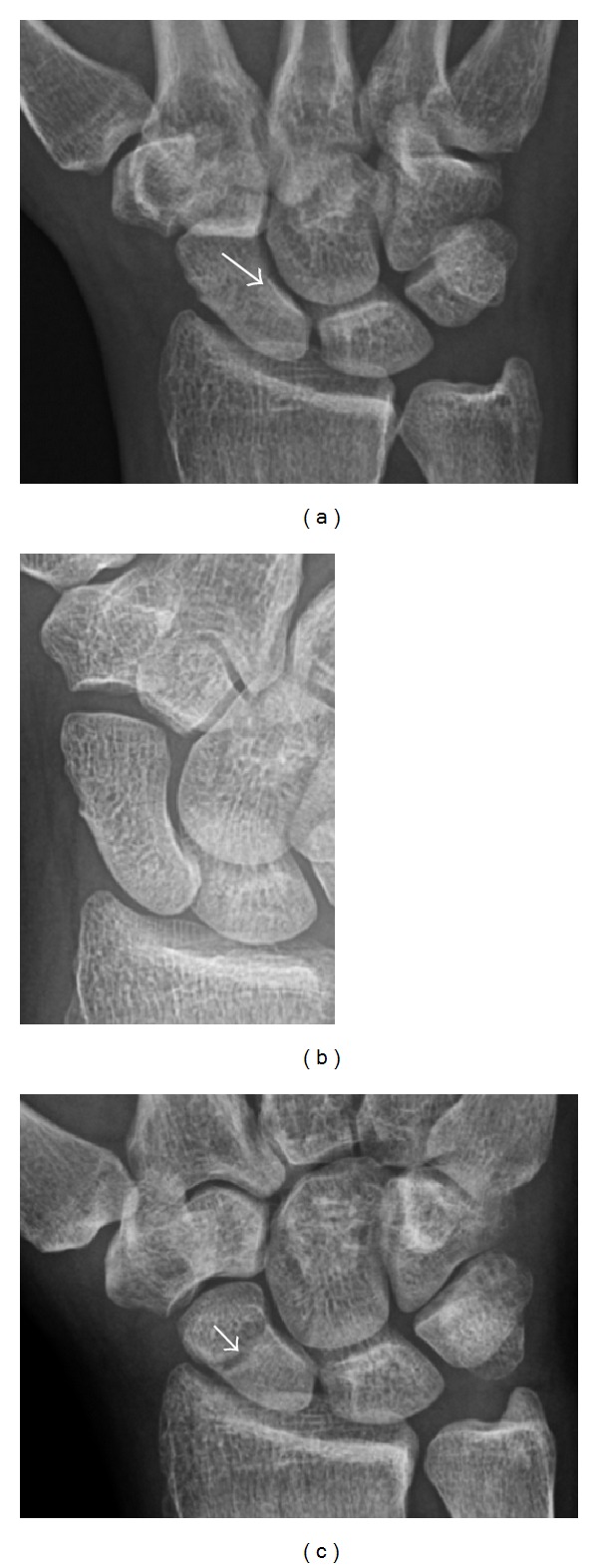
A 26-year-old man presenting with wrist pain after being assaulted. (a) Initial anteroposterior radiograph shows a subtle linear lucency within the scaphoid extending to the scaphocapitate articular surface that was overlooked (arrow). (b) Initial “scaphoid” view was negative. (c) Followup anteroposterior radiographs, 12 days later, shows obvious scaphoid fracture (arrows).

Occult osseous injuries may result from a direct blow to the bone by compressive forces of adjacent bones against one another or by traction forces during an avulsion injury. Lesions in the tibial plateau, hip, ankle, and wrist are often missed [37]. In a tibial plateau fracture, any disruption of the posterior and anterior cortical rims of the plateau should be sought. Impaction of subchondral bone will appear as an increased sclerosis of the subchondral bone (Figure 1). In the hip, posterior acetabular fractures also present subtle radiographic findings. The acetabular lines should then be carefully examined keeping in mind that the posterior rim, which is harder to see on X-rays, is more frequently fractured than the anterior rim (Figure 2). In the wrist, detection of carpal bone fractures is often challenging, with up to 18% of scaphoid fractures radiographically occult [38]. Carpal fractures, especially the scaphoid, are associated with the risk of avascular necrosis [38]. In apparently normal wrist radiographs from symptomatic patients, if there is history of a fall on an outstretched hand with pain in the anatomic snuffbox, suggesting scaphoid injury, the initial examination with posteroanterior, lateral, and pronation oblique views must be complemented by other specific views such as supination oblique and the “scaphoid” view [3]. A careful examination of cortices for evidence of discontinuity or offset and cancellous bone for lucency is necessary (Figure 3). 

**Figure 4:**
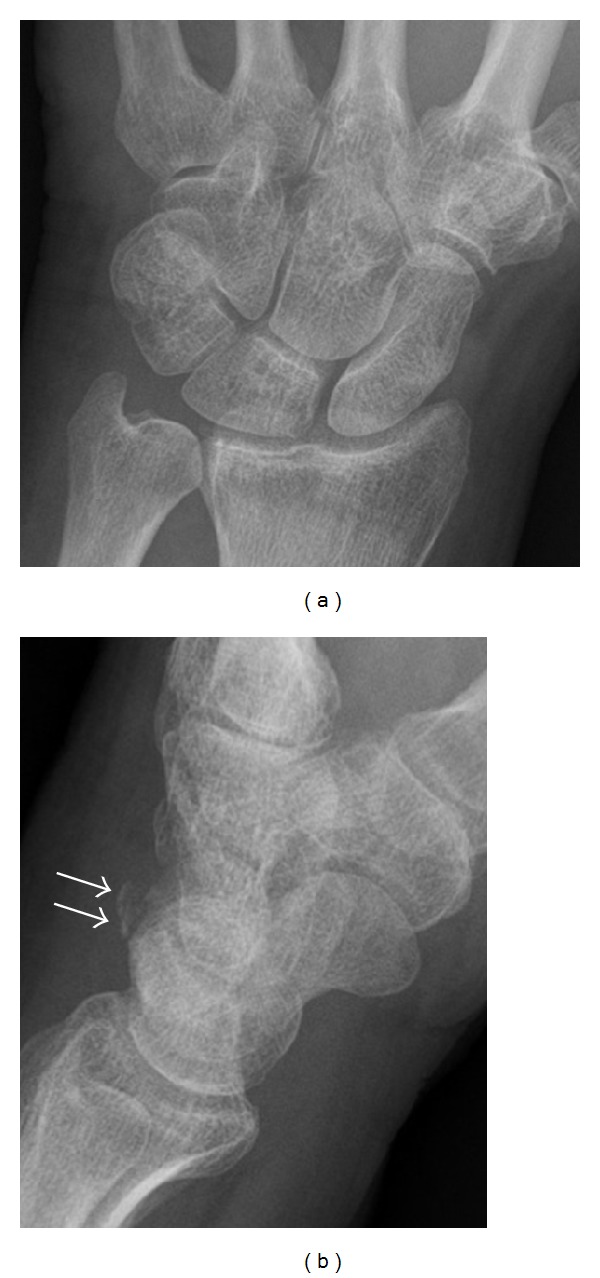
Dorsal triquetral fracture of the left wrist in a 30-year-old man after a trauma. Anteroposterior radiograph shows a normal appearance. (b) Lateral radiograph of the same wrist demonstrates a chip fracture off the dorsal aspect of the triquetrum (arrow).

Triquetral fracture usually occurs on the dorsal aspect by impingement from the ulnar styloid or avulsion of strong ligamentous attachment [3]. The dorsal avulsion fracture or “chip fracture” appears as a small bony fragment on the dorsal aspect of the triquetrum and is best detected on the lateral view [3] (Figure 4). When radiography is negative in patients with high suspicion of a fracture, both MRI and MDCT will be of value [39]. However, it has been shown that MRI is superior for detecting trabecular fractures in carpal bones [16]. 

**Figure 5:**
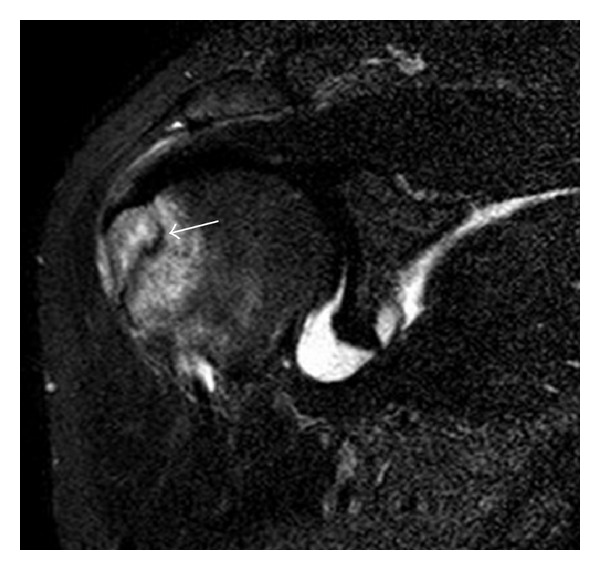
Traumatic fracture of the greater tuberosity in a 51-year-old man presenting with left shoulder pain after a fall on ice. Initial radiographs were normal. Coronal inversion recovery MRI shows a fracture line (arrow) through the greater tuberosity surrounded by a bone marrow edema pattern.

**Figure 6:**
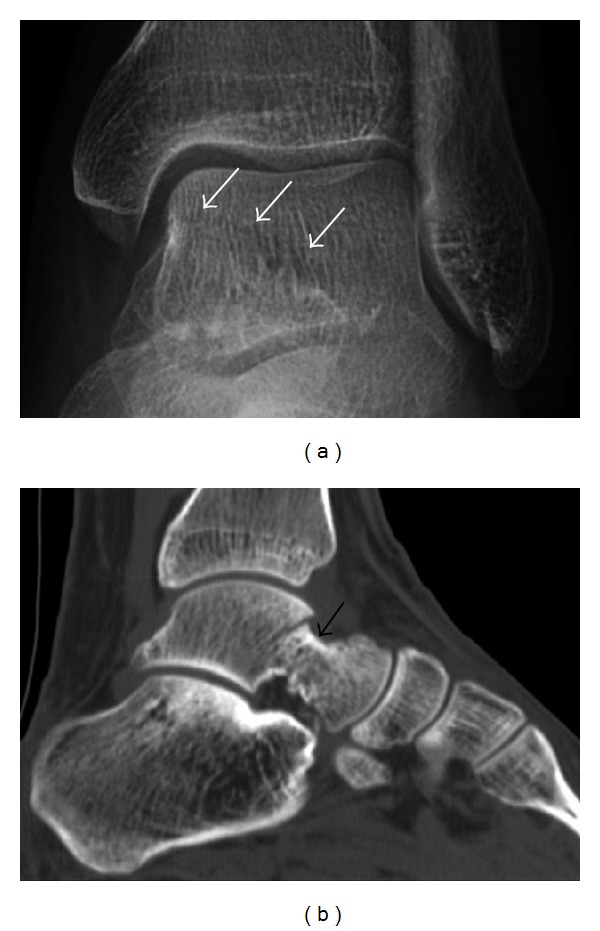
Subtle anterior talar fracture in a 39-year-old man presenting with ankle pain after a fall. (a) Anteroposterior radiograph shows a subtle oblique radiolucent line through the talus (white arrows). (b) Sagittal CT reformation confirms the presence of an anterior talar fracture with cortical offset (black arrow).

The greater tuberosity of the humerus is also an illustrative location of occult fractures. The osseous injury may follow seizures, glenohumeral dislocation, forced abduction, or direct impaction. They are commonly discovered on MRI in symptomatic patients with suspicion of rotator cuff tear. Coronal images are best suited for detection. They appear as crescentic oblique lines surrounded by a bone marrow edema pattern (Figure 5). The rotator cuff must be inspected since associated ligamentous lesions are common. In the ankle, malleoli and tarsal bones should be checked carefully for any cortical disruptions and radiolucent lines that may reveal a fracture. Awareness of the exact location of the pain will help direct the attention of the interpreter when searching for very subtle signs of fracture (Figure 6). 

Avulsion fractures, which consist of a detached bone fragment resulting from a ligament or tendon pulling away from the bone, may also present with subtle radiographic sign**s**. Tiny osseous fragments near the presumed attachment site of a ligament suggest this diagnosis. Common sites are the lateral tibial plateau (the Segond fracture), the spinal tuberosity of the tibia resulting from anterior cruciate ligament avulsion, and the ischial tuberosity.

## 4. Fatigue Fractures

Fatigue fractures occur when healthy bone is exposed to repeated stress. The bone is a living tissue, with the capacity to repair itself; fatigue fractures occur when repetitive injuries exceed the repair capacity of the bone [14]. This type of fracture does not occur as a single event but rather incrementally as a sequence of cellular events that begin with increased osteoclastic activity [14, 40]. Microfractures occur later and are accompanied by bone marrow edema, which can be detected on MRI. This stage appears on MRI as an isolated bone marrow edema pattern without a fracture line and is called stress reaction. Then, periosteal new bone forms and may be visible on radiography. Full cortical fractures occur if the repetitive stress continues. Only timely detection and appropriate management can interrupt this sequence. 

**Figure 7:**
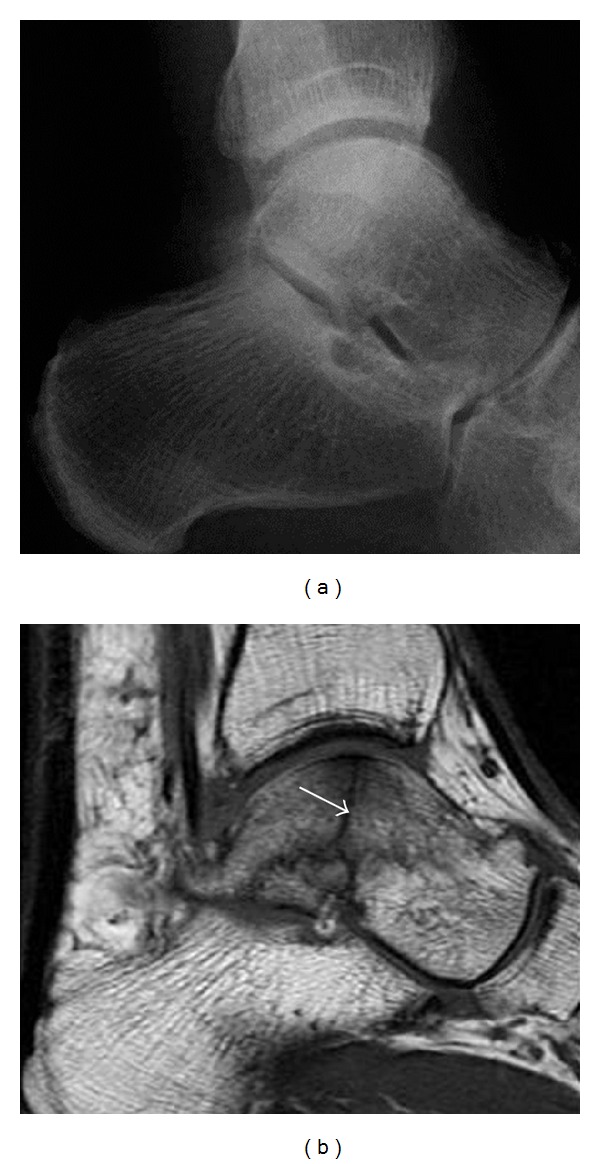
Fatigue fracture of the talus in a 25-year-old male basketball player with right hind foot and ankle pain, without history of trauma, and a normal initial radiograph (not shown). (a) One-month followup lateral radiograph shows normal appearance. (b) Sagittal T1-weighted MRI shows an irregular fracture line (arrow) within an ill-defined area of hypointensity corresponding to bone marrow edema.

**Figure 8:**
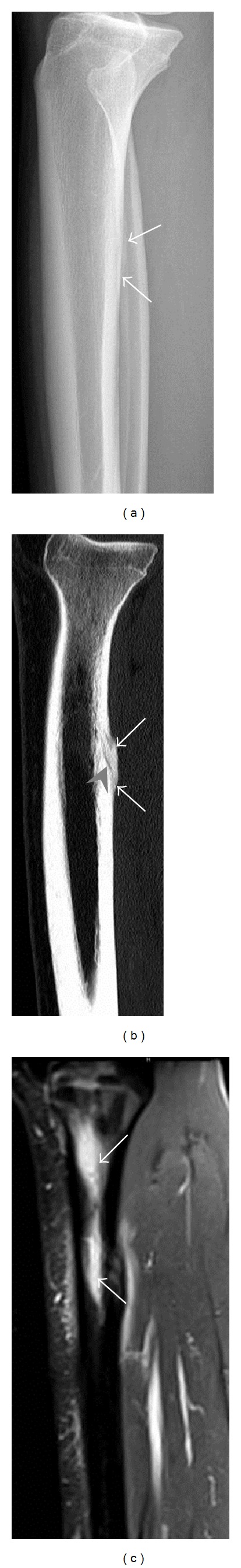
Proximal diaphyseal fatigue fracture of the tibia in a 20-year-old man with a history of regular jogging. (a) Lateral radiograph shows no obvious fracture lines but a subtle localized medial tibial cortex periosteal reaction (arrows). (b) Sagittal reformatted CT image acquired 1-month after the radiograph shows a linear hypoattenuation in the tibial cortex (arrowhead), as well as obvious periosteal thickening (arrows). (c) Sagittal T2-weighted fat-saturated image acquired the same day shows an area of hyperintensity spreading over the proximal tibia (arrows), which is consistent with the presence of proximal tibial fracture.

**Figure 9:**
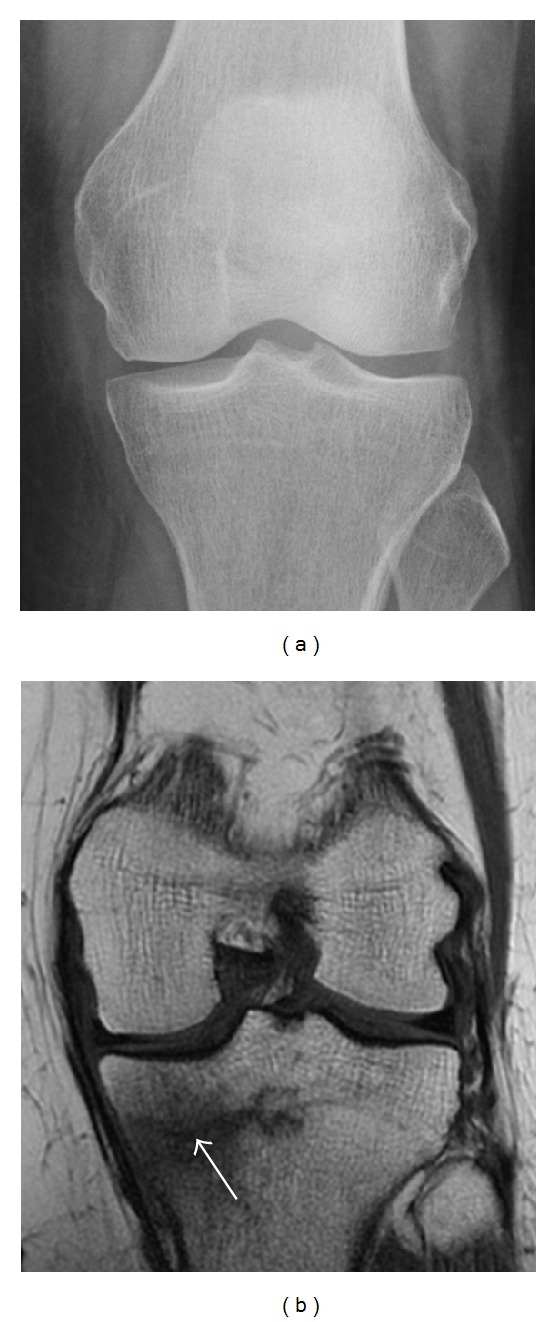
Proximal metaphyseal fatigue fracture of the tibia in a 27-year-old recent male military recruit. (a) Anteroposterior radiograph is within normal limits. (b) Coronal T1-weighted MR image shows a marked linear hypoattenuation along the medial tibial metaphysis (arrow) surrounded by diffuse hypointensity in keeping with posttraumatic edema.

**Figure 10:**
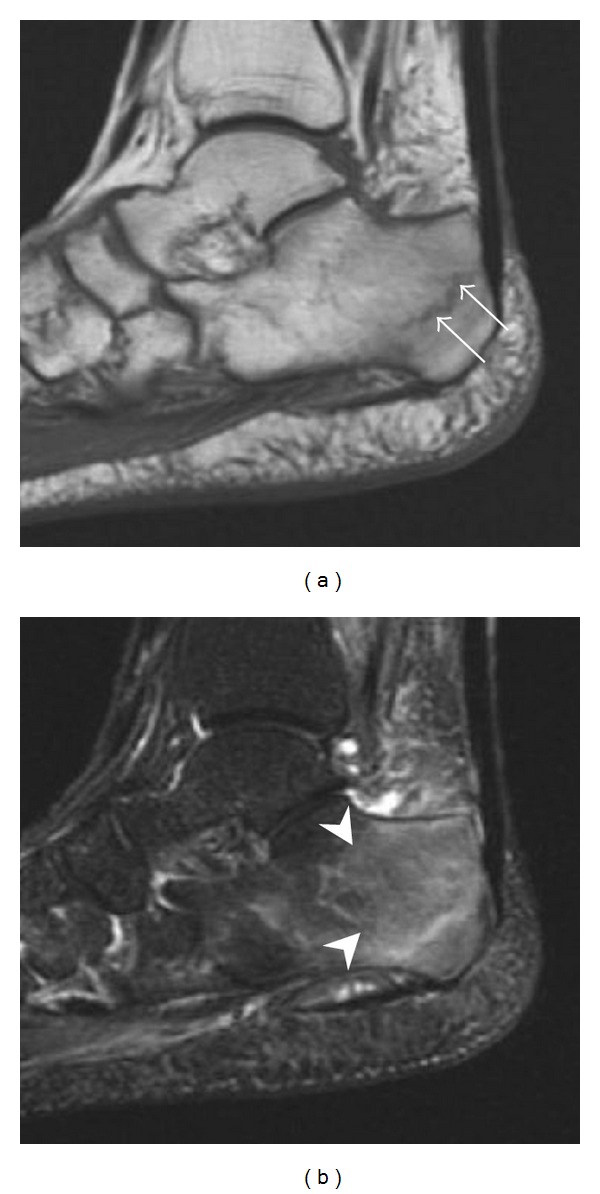
Calcaneal fatigue fracture in a 30-year-old male runner. Radiographs were normal (not shown). (a) Sagittal T1-weighted and (b) short tau inversion recovery images show a linear hypointensity (arrows) of calcaneal tuberosity within diffuse bone marrow edema, which appears as an ill-defined area of hyperintensity on a fluid sensitive pulse sequence (arrowheads).

**Figure 11:**
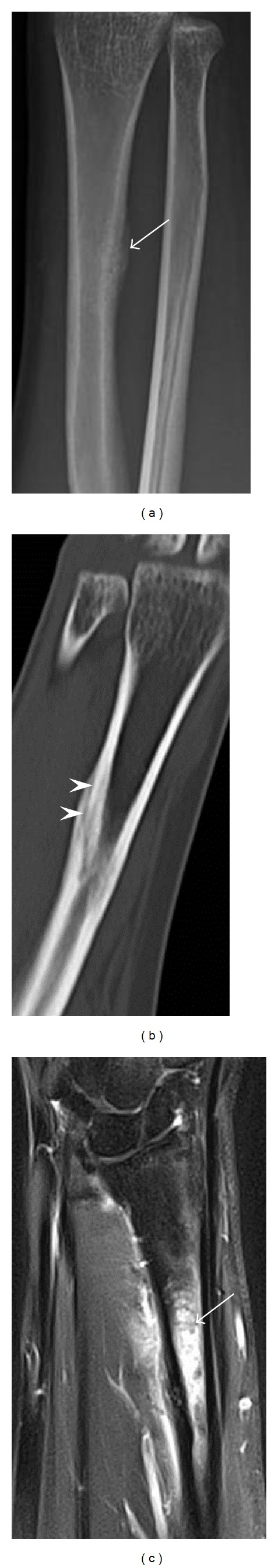
Stress fracture of the right radius in a 40-year-old man, a semiprofessional billiard player, with no history of trauma and complaining of pain of the right forearm for one month. (a) Anteroposterior radiograph shows medial radial cortex periosteal reaction (arrow) but no fracture line is seen. (b) Coronal reformatted CT depicts monocortical fracture line through the periosteal thickening (arrowheads). (c) Coronal T2-weighted fat-suppressed MRI shows intramedullary hyperintensity within the bone marrow (arrow) corresponding to bone marrow edema.

Fatigue fractures are more frequent in women which may be due to the relatively smaller bones of women. Moreover, pregnancy is a well-recognized risk factor for femoral neck fatigue fracture. While fibular and metatarsal fractures have a low risk of complications, other sites including the femoral neck, midanterior tibia, navicular, talar, and other intraarticular fractures are prone to complications such as delayed union, nonunion, and displacement [14]. The site of the insufficiency fracture may be specific to the activity: for example, rugby and basketball players are more prone to navicular fractures, while gymnasts have a higher risk for talar fractures (Figure 7). Long distance runners are at increased risk for pelvic, tibial (Figures 8 and 9), and fibular fractures [14]. In the military, calcaneus (Figure 10) and metatarsals are the most commonly cited injuries, especially in new recruits [15]. Billiard players are at risk for upper limb fractures (Figure 11).

Radiographic examination usually shows delayed signs of fracture up to 2 to 3 months after initial injury. In a bony region with a high proportion of cancellous bone (e.g., femoral neck), a fatigue fracture appears as an ill-defined transverse sclerotic band (in contact or close to the medial cortex), with a periosteal thickening appearing at a later stage. In case of continued stress, a fracture line through the thickened cortex and a region of sclerosis may be observed [6]. MRI is of great value for early diagnosis and displaying bone marrow edema, while scintigraphy is useful for showing increased metabolic activity within the bone. However, MRI is preferred since scintigraphy lacks specificity. In case of isolated bone marrow edema in MRI without a fracture line, the diagnosis of fatigue fracture may be more complicated, and other conditions such as transient edema and osteoid osteoma need to be excluded [14]. Additional imaging by CT is warranted in such cases.

## 5. Insufficiency Fractures

Insufficiency fractures occur in weakened bones. Although osteoporosis is a classic cause, other conditions resulting in bone demineralization are well-recognized risk factors. These include previous radiation therapy and chemotherapy, especially in a context of gynaecologic malignancy, chronic renal failure, chronic rheumatological diseases, and corticosteroid therapy [24]. In long bones, chronic joint diseases such as rheumatoid arthritis are associated with angular deformity and flexion contraction, increasing the stress on the bone around the joints [41] and, therefore, the risk of insufficiency fracture. Pelvic, sacral, and proximal femoral fractures are of increasing significance especially with the aging of the population [42]. 

**Figure 12:**
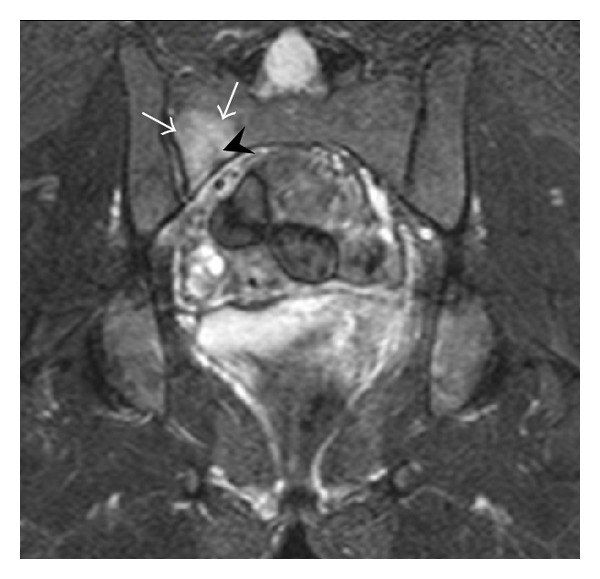
Right sacral alar insufficiency fracture in a 29-year-old woman with a 9-year history of corticosteroid therapy for systemic lupus erythematous. Conventional radiographs showed normal appearance (not shown). Coronal inversion recovery MRI shows an area of hyperintensity in the right sacral ala (white arrows), centered on a linear hypointensity corresponding to the fracture line (black arrowhead).

The sacrum is usually masked by overlapping bowel gas in conventional radiographs, and the subtle radiographic findings are usually nondiagnostic and even misleading. The characteristic “H” pattern has been correlated with biomechanical models of patient activities. The vertical parasagittal planes correspond to the region of maximal stress during walking, while the horizontal fracture develops later, secondary to the loss of lateral support by parasagittal fractures [43]. MRI is the primary imaging technique in this case, with the most common MRI pattern showing bone marrow edema and a fracture line [18] (Figure 12). Coronal views are quite contributive in sacral fractures, allowing the detection of the horizontal component, especially with fluid-sensitive sequences. Although the sacrum is the most commonly involved, pelvic insufficiency fractures are often multiple, and other typical locations should be mentioned. 

**Figure 13:**
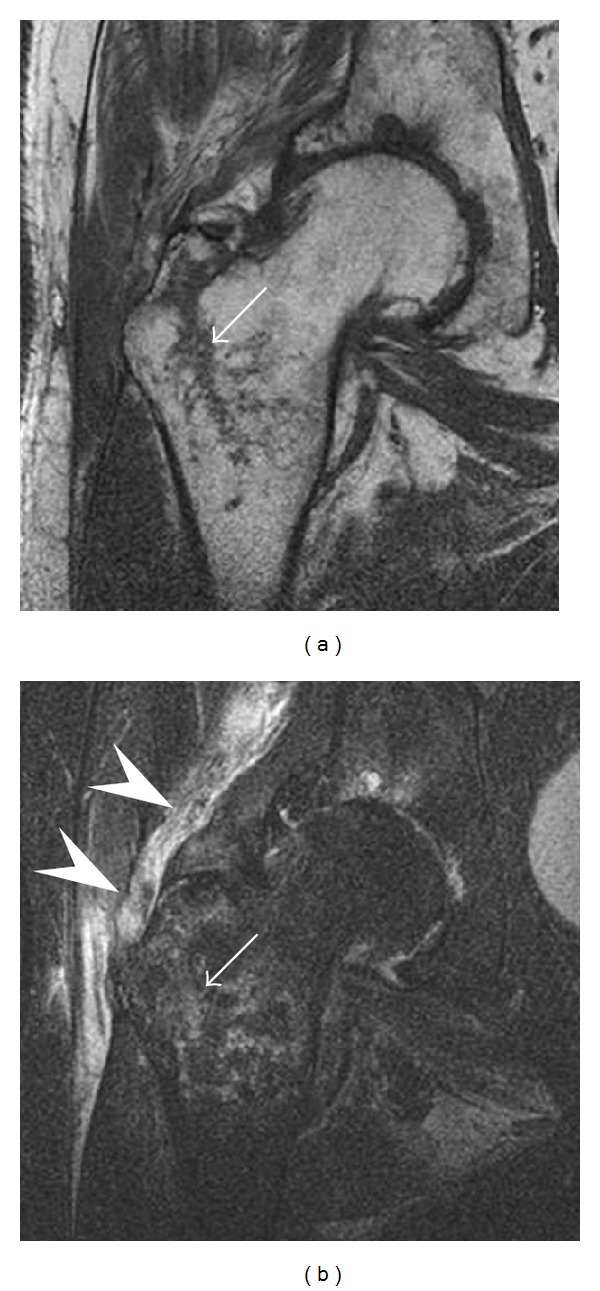
Partial osseous avulsion of the gluteal muscles at the greater trochanter in a 59-year-old man who presented with the right hip pain without a history of trauma. Lauenstein view and anteroposterior and radiographs (not shown) did not show an obvious fracture line or disruption of bony contours in the acetabulum or the right femoral neck. (a) Coronal T1-weighted MRI displays an incomplete fracture line extending partially from the greater trochanter (arrow). (b) Coronal short tau inversion recovery MRI shows heterogeneous hyperintensity in the same region (arrow) as well as hyperintensity within the gluteus medius and minimus muscles (arrowheads) consistent with tissue edema and hematoma.

**Figure 14:**
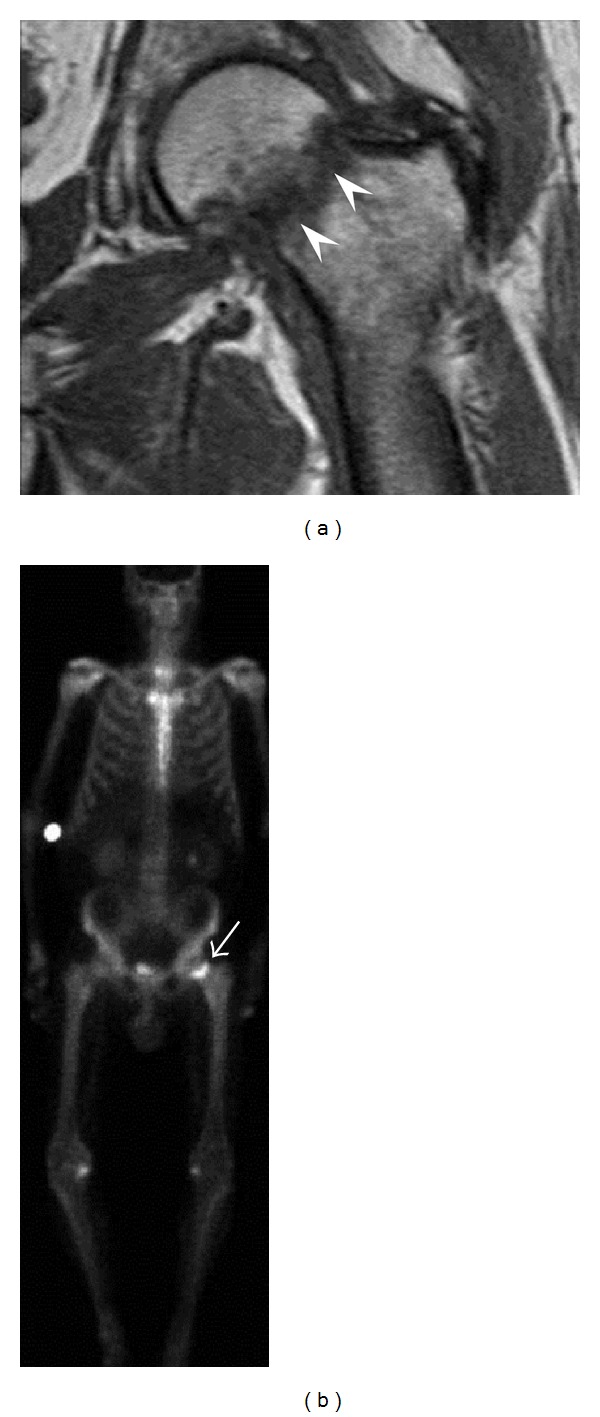
Subcapital insufficiency fracture in a 55-year-old man with a left hip pain without a history of trauma. Anteroposterior and Lauenstein view radiographs centered on the left hip do not show an obvious fracture line, but mild acetabular osteophytosis was noted consistent with hip osteoarthritis (not shown). (a) Coronal T1-weighted MRI shows a linear low-signal band through the femoral neck corresponding to a fracture line (arrowheads). (b) Bone scintigraphy shows focal uptake (arrow) corresponding to the fracture.

Proximal femoral fractures usually occur in osteoporotic patients, and their signs include subtle neck angulation, trabecular angulation, and subcapital impaction line. A frog-leg lateral view may be helpful if the greater trochanter is short enough. However, positioning can be difficult because of hip pain. In patients with strong suspicion of proximal femoral fracture and negative radiographs, MRI limited to coronal T1 W images and scintigraphy can be highly valuable (Figures 13 and 14). Such an option, with limited examination time, is cost-effective and allows reliable exclusion or confirmation of the diagnosis, preventing an unnecessary stay at the hospital or delayed treatment [23, 25]. Moreover, MRI helps to detect soft tissue abnormalities which are more frequently seen in femoral, acetabular, and pubic injuries than sacral lesions. Concomitant fractures are also frequently seen in typical pelvic sites [18]. 

## 6. Conclusion

Radiographically occult and subtle fractures are often a challenging diagnostic problem in daily clinical practice. Radiologists should be aware of the different situations and mechanisms of these injuries as well as the subtle radiographic signs that can be encountered in each situation. The knowledge of normal images and the consideration of the clinical context are of great value in improving the detection of these fractures either on conventional radiographs or with more advanced imaging tools.
